# Gemigliptin Improves Salivary Gland Dysfunction in D-Galactose-Injected Aging Rats

**DOI:** 10.3390/pharmaceutics16010035

**Published:** 2023-12-26

**Authors:** Woo Kwon Jung, Su-Bin Park, Hwa Young Yu, Junghyun Kim

**Affiliations:** Department of Oral Pathology, School of Dentistry, Jeonbuk National University, Jeonju 54896, Republic of Korea; woo501@jbnu.ac.kr (W.K.J.); tnqls309@gmail.com (S.-B.P.); enzks17@gmail.com (H.Y.Y.)

**Keywords:** gemigliptin, D-galactose, hyposalivation, salivary hypofunction, anti-glycation, aging

## Abstract

Oral dryness is among the most common conditions experienced by the elderly. As saliva plays a crucial role in maintaining oral health and overall quality of life, the condition is increasingly taking its toll on a rapidly growing aging population. D-galactose (D-gal) stimulates their formation, which in turn cause oxidative stress and accelerate age-related decline in physical function. In this study, we observed a reduction in salivary secretion and amylase levels in aged rats injected with D-gal, confirming salivary gland dysfunction. Treatment with gemigliptin increased DPP-4 inhibition and GLP-1 levels in the salivary glands of aging rats and reduced the expression of AGEs and receptors for advanced glycation end products (RAGE). This effect was caused by the presence of additional reactive oxygen species (ROS) in the salivary glands of the examined rats. Gemigliptin’s cytoprotective effect reduced amylase and mucin accumulation and increased AQP5 expression, which are important indicators of salivary gland function. In sum, gemigliptin was shown to improve D-gal-induced decline in the salivary gland function of aged rats through its anti-glycation and antioxidant activities. Gemigliptin shows promise as a treatment strategy for patients experiencing decreased salivary function associated with their advancing age.

## 1. Introduction

Aging is a progressive biological process characterized by the deterioration of bodily functions over time. The proportion of the global population that is elderly is increasing, and it is estimated to reach 22% by 2050 [1]. Salivary hypofunction is among the most common issues affecting the elderly [2]. Decreased salivary flow and changes to saliva composition can lead to more dental caries, susceptibility to oral candidiasis, oral dryness, speech difficulties, swallowing problems, altered taste perception, and halitosis [3]. One of the causes of dry mouth in the elderly is salivary hypofunction, which is caused by changes in acinar cells [4]. Previous reports have observed average decreases of 38.5% in salivary flow rate and 38.0% in stimulated salivary flow in older adults [5]. Numerous studies have investigated the effects of aging on salivary gland secretion, but the exact relationship is still unclear, with further research needed.

Advanced glycation end products (AGEs), which accumulate as a result of aging, are a heterogeneous and complex group of compounds formed through a non-enzymatic Maillard reaction between reduced sugars and amino acids in proteins and other macromolecules [6]. AGEs can be produced both exogenously and endogenously. Research on AGE in the body has linked them to certain chronic diseases, such as diabetes and the aging process itself [7]. The accumulation of AGEs accelerates age-related functional decline. AGEs have detrimental effects on virtually all cells, tissues, and organs [8].

Accumulation of AGEs in the salivary glands of D-galactose (D-gal)-induced aging rats has been shown to result in increased generation of reactive oxygen species (ROS), loss of acinar cells, and an increase in cell apoptosis [9]. Additionally, D-gal-induced aging mice have exhibited decreased aquaporin levels, inflammation, and induction of cell apoptosis [10]. A previous study on aging rats showed that salivary hypofunction was improved by the antioxidant and anti-glycation effects of regular physical exercise [9]. Another study showed that polydatin improved salivary gland hypofunction in an experimental animal model of diabetes through its antioxidant and anti-glycation activities [11,12].

Dipeptidyl peptidase-4 (DPP-4) inhibitors are a relatively new class of oral antidiabetic drugs that are already widely used in various combination therapies (Table 1). These regulate blood sugar levels by preventing the inactivation of the incretin hormone Glucagon-like peptide-1 (GLP-1) [13]. Gemigliptin (Gemiglo^®^, LG Life Sciences, Daejeon, Republic of Korea) is a DPP-4 inhibitor approved for use in patients with type 2 diabetes [14]. DPP-4 inhibitors have been reported to have cytoprotective effects against various types of organ damage, not just glucose regulation [15]. The DPP-4 inhibitor saxagliptin, for example, suppressed increased insulin signaling impairment and oxidative stress in a D-gal-induced aging model [16]. The anti-glycation effects of DPP-4 inhibitors have been well documented. Linagliptin treatment improves diabetic retinopathy by reducing oxidative stress and decreasing AGE formation [17]. Linagliptin also reduces AGE-mediated oxidative stress in cultured endothelial cells and lowers AGE levels, RAGE expression, and oxidative stress in diabetic rats [18]. Vildagliptin prevents diabetes-related vascular damage by reducing AGEs, RAGE, and oxidative marker levels [19]. Recently, the DPP-4 inhibitor alogliptin was found to block the AGE-RAGE axis, and consequently reduce albuminuria in patients with type 2 diabetes [20]. Gemigliptin’s anti-glycation effects have been validated through the capture of methylglyoxal and inhibition of AGEs as well as through protein cross-linking reactions observed in in vitro and in vivo experiments [21]. Gemigliptin has a superior DPP-4 inhibitory effect compared to other gliptin-based drugs that are DPP-4 inhibitors, and it offers several advantages when taken [22]. Additionally, our previous research validated its ability to suppress salivary dysfunction in streptozotocin-induced diabetic rats [23]. Building on these findings, this study aimed to verify the effectiveness of gemigliptin at improving salivary gland dysfunction caused by aging.

## 2. Materials and Methods

### 2.1. Animals

Six-week-old male Sprague-Dawley (SD) rats were obtained from Damul Science (Daejeon, Republic of Korea). Salivary gland dysfunction was induced by administering D-gal at a dosage of 300 mg/kg for a period of 4 weeks. Additionally, gemigliptin along with D-gal was administered orally once a day for 4 weeks. Rats were acclimatized to the environment for one week and then randomly divided into five groups: Group 1: normal rats (Nor, *n* = 7); Group 2: rats treated with D-gal (300 mg/kg) via intraperitoneal (i.p.) injection (D-gal, *n* = 7); Group 3: rats treated with D-gal (300 mg/kg) via i.p. injection and orally administered 100 mg/kg of aminoguanidine (AG), a well-known anti-glycation agent (AG, *n* = 7); Group 4: rats treated with D-gal (300 mg/kg) via i.p. injection and orally administered 50 mg/kg of gemigliptin (GG10, *n* = 7); Group 5: rats treated with D-gal (300 mg/kg) via i.p. injection and orally administered 100 mg/kg of gemigliptin (GG100, *n* = 7). D-gal was administered once a day for 4 weeks. All procedures involving animals were approved by the Institutional Animal Care and Use Committee of the Jeonbuk National University Laboratory Animal Center (IACUC, approved no.: JBNU 2020-0102).

### 2.2. Collection of Saliva

The rats were anesthetized, and pilocarpine hydrochloride (1.5 mg/kg, IP) was administered. Cotton balls were placed in the mouths of the rats to collect saliva for 15 min. After 15 min, the cotton balls were weighed to determine the amount of saliva absorbed. Salivary secretion was compared and analyzed by calculating the difference in weight between the cotton balls before and after saliva absorption.

### 2.3. Western Blot Analysis

Protein analysis was performed using protein lysates from cells and tissues. Proteins were separated by SDS-PAGE gel and then transferred to PVDF membrane, first blocked with 5% skim milk and then incubated overnight at 4 °C with specific antibodies as follows. Bax rabbit monoclonal antibody (1:1000, Abcam, Waltham, MA, USA), Bcl-2 mouse monoclonal antibody (1:1000, Enzo, Beverly, MA, USA), caspase-3 rabbit monoclonal antibody (1:1000, Cell Signaling Technology, Beverly, MA, USA), caspase-9 mouse monoclonal antibody (1:1000, Cell Signaling Technology, Beverly, MA, USA), β-actin mouse monoclonal antibody (1:2000, Santa Cruz Biotechnology, Santa Cruz, CA, USA), catalase rabbit monoclonal antibody (1:2000, Cell Signaling Technology, Beverly, MA, USA), SOD1 rabbit monoclonal antibody (1:2000, Cell Signaling Technology, Beverly, MA, USA), AGE mouse monoclonal antibody (1:1000, TransGenic, Inc., Fukuoka, Japan), and aquaporin 5 (AQP5) rabbit monoclonal antibody (1:1000, Bioworld Technology, Louis Park, MN, USA). After washing three times (15 min each) with TBST buffer (10 mM Tris-HCl, pH 7.4, 150 mM NaCl, and 0.1% Tween-20), we incubated the membranes with Horseradish peroxidase-conjugated anti-rabbit and anti-mouse IgG secondary antibodies (1:2000, Enzo, Beverly, MA, USA) at room temperature for 1 h. Following this, we rinsed the membranes with TBST buffer and then detected the signals using WesternBright ECL (Advansta Inc., Menlo Park, CA, USA). Protein expression levels were quantified using an image analyzer (ATTO Corporation, Tokyo, Japan). All protein expression levels were normalized to the expression of β-actin.

### 2.4. Apoptosis Analysis by TUNEL Staining

We performed a cell apoptosis analysis within the salivary gland tissue of rats. The samples were fixed with formaldehyde, followed by dehydration and embedding in paraffin blocks. We then sectioned the paraffin blocks and deparaffinized the tissue sections twice in xylene (each time for 5 min), which were then rehydrated in ethanol. A terminal deoxynucleotidyl transferase dUTP nick-end labeling (TUNEL) assay was performed according to the instructions associated with the DeadEndTM Fluorometric TUNEL System from Promega (Madison, WI, USA). Cell nuclei were counterstained with DAPI mounting solution (4’,6-diamidino-2-phenylindole, Santa Cruz Biotechnology, Santa Cruz, CA, USA), and TUNEL-positive cells were quantified. TUNEL-positive apoptotic cells were visualized using green fluorescence emitted by a BX51 microscope (Olympus, Tokyo, Japan). Five high-power fields (×200) were randomly selected for each group, and the number of TUNEL-positive cells was calculated using ImageJ software v1.53k (NIH, Bethesda, MD, USA).

### 2.5. Histopathological Examination

The 4 μm thick sections, fixed in 10% formalin and embedded in paraffin, were deparaffinized and rehydrated. Hematoxylin and Eosin (H&E) staining was performed for histological evaluation. The ratio of the surface area occupied by acinar cells and ductal cells in the salivary glands was assessed using light microscopy at a magnification of ×100. Three different fields from each of the five (*n* = 5) sections of salivary glands were analyzed using ImageJ software v1.53k. To assess the accumulation of neutral mucins (neutral mucopolysaccharides dyed pink), we stained the sections with Periodic Acid-Schiff (PAS) and performed a counterstain with hematoxylin. To provide a more detailed comparison, we also presented the PAS staining results without the hematoxylin counterstain. To assess the accumulation of acidic mucins (acid mucopolysaccharides dyed blue), we stained the sections with Alcian blue 2.5 pH and counterstained with Nuclear Fast Red. Slide observations were made using an optical microscope (BX51, Olympus, Tokyo, Japan).

### 2.6. Immunohistochemistry

The 4 μm thick sections, initially fixed in 10% formalin and subsequently embedded in paraffin, underwent deparaffinization and rehydration through a gradient of ethanol. Antigen retrieval was performed by subjecting the sections to Tris-EDTA Buffer (10 mM Tris Base, 1 mM EDTA Solution, 0.05% Tween 20, pH 9.0) using a microwave. Endogenous peroxidase activity was neutralized with 3% hydrogen peroxide, followed by a 30 min blocking step using 2.5% normal horse serum. The following were used as primary antibodies: 8-hydroxy-2’-deoxyguanosine (8-OHdG 1:1000, Abcam, Waltham, MA, USA), High mobility group box 1 (HMGB1, 1:1000, Cell Signaling Technology, Beverly, MA, USA), AGE (1:500, TransGenic, Inc., Fukuoka, Japan), RAGE (1:500, Santa Cruz Biotechnology, Santa Cruz, CA, USA), and AQP5 (1:500, Bioworld Technology, Louis Park, MN, USA). Signals for the primary antibodies were detected using the VECTASTAIN Elite ABC Universal Kit (Vector Laboratory, Burlingame, CA, USA), and visualization was achieved with the DAB peroxidase substrate kit (Vector Labs, Burlingame, CA, USA). Counterstaining was performed with hematoxylin, after which the samples were mounted. As a negative control group, tissue sections were incubated with non-immune animal serum instead of the primary antibodies. Observations were made using an optical microscope (BX51, Olympus, Tokyo, Japan). Quantification was carried out by measuring the average optical density (mm^2^) per unit area in five randomly selected areas at 200× magnification using ImageJ software v1.53k (NIH, Bethesda, MD, USA).

### 2.7. Measurement of DPP-4 and GLP-1

The DPP-4 activity and levels of DPP-4 and Glucagon-like peptide 1 (GLP-1) were measured using the rat GLP-1 ELISA Kit (MyBioSource, San Diego, CA, USA) and rat DPP-4 ELISA kit (MyBioSource, San Diego, CA, USA), respectively, following the manufacturer’s instructions. The results were measured using the Spark^®^ Multimode Microplate Reader (Tecan, Männedorf, Switzerland).

### 2.8. Measurement of Amylase Alpha 1

Following the manufacturer’s instructions, we detected amylase secretion in saliva using the rat amylase alpha 1, salivary ELISA Kit (MyBioSource, San Diego, CA, USA). Optical density measurements were taken using a Spark^®^ Multimode Microplate Reader (Tecan, Männedorf, Switzerland).

### 2.9. Measurement of AGEs

Following the manufacturer’s instructions, we determined the levels of AGEs in salivary gland tissue, saliva, and serum using the rat AGEs ELISA kits (MyBioSource, San Diego, CA, USA). Optical density measurements were taken using a Spark^®^ Multimode Microplate Reader (Tecan, Männedorf, Switzerland).

### 2.10. Statistical Analysis

Statistical analysis was performed using one-way analysis of variance (ANOVA) followed by Tukey’s multiple comparison test between groups using Prism 8.0 software (GraphPad, San Diego, CA, USA).

## 3. Results

### 3.1. Gemigliptin Prevented D-Gal-Induced Salivary Gland Dysfunction and Pathological Changes

**Figure 1:**
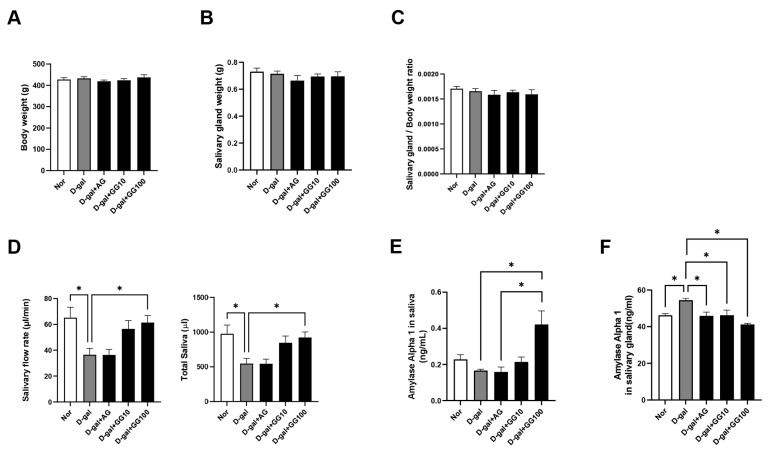

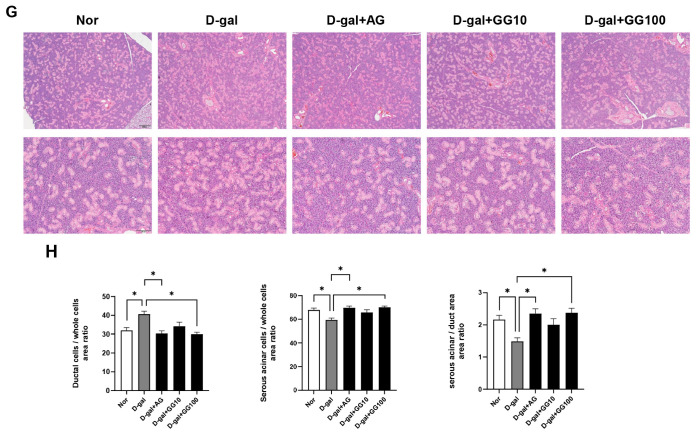
Gemigliptin (GG) ameliorates salivary gland dysfunction and pathological alterations in D-galactose (D-gal)-injected rats. (**A**) Body weight in each group. (**B**) Salivary gland weight in each group. (**C**) Salivary gland weight divided by body weight. (**D**) Saliva secretion was induced by intraperitoneal injection of pilocarpine, and total saliva secretion was measured for 15 min. The levels of amylase were measured using an ELISA kit. (**E**) Measurement of amylase concentration in collected saliva. (**F**) Measurement of amylase concentration in salivary gland lysate. (**G**) Representative salivary glands stained with H&E (scale bar = 100 μm). (**H**) Quantitative evaluation of salivary glands acinar cell area and duct area. All data are presented as the mean ± standard error of the mean (*n* = 5). * *p* < 0.05.

An in vivo experiment was conducted to investigate the effects of gemigliptin induced by D-gal on salivary gland dysfunction in aging rats. There were no significant differences in body weight and salivary gland weight among the groups (Figure 1A–C). However, in the experiment measuring salivary secretion, it was observed that the salivary flow rate had been significantly reduced in the D-gal group relative to the control group, while the gemigliptin 100 mg/kg group showed a significant increase (Figure 1D). The concentration of amylase in the saliva decreased in the D-gal group and showed a dose-dependent increase in the groups treated with gemigliptin. Moreover, the gemigliptin 100 mg/kg group exhibited a significant increase in amylase concentration compared to the AG group (Figure 1E). Conversely, the concentration of amylase in the salivary gland increased in the D-gal group but decreased in the gemigliptin-treated groups (Figure 1F). To observe morphological changes in the salivary glands, we performed H&E staining. The study examined the ratio of the ductal area to the serous acinar cell area and determined that the D-gal group had an increased ductal cell area and a decreased serous acinar cell area. In contrast, the gemigliptin-related group showed a decrease in ductal cell area and an increase in the serous acinar cells area (Figure 1G,H). In conclusion, aging induced by D-gal resulted in decreased salivary secretion, changes in amylase concentration in both the saliva and salivary glands, and morphological alterations to the salivary glands. The administration of gemigliptin improved these effects.

### 3.2. Effect of Gemigliptin on DPP-4 Activity and GLP-1 Levels in the Salivary Gland and Serum

**Figure 2:**
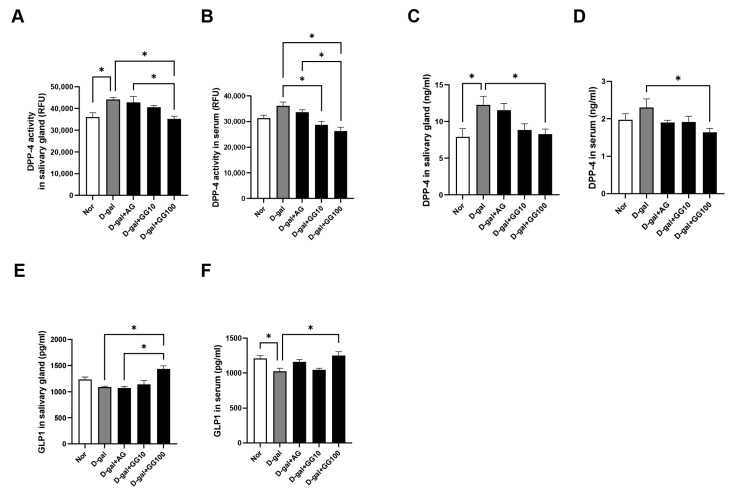
Effects of GG on DPP-4 and GLP-1 activity and levels in salivary glands and serum of D-gal-injected rats. The DPP-4 activity and levels of DPP-4 and GLP-1 were measured using an ELISA kit. (**A**) DPP-4 activity in salivary glands. (**B**) DPP-4 activity in serum. (**C**) Levels of DPP-4 in salivary glands. (**D**) Levels of DPP-4 in serum. (**E**) Levels of GLP-1 in salivary glands. (**F**) Levels GLP-1 in serum. All data are presented as the mean ± standard error of the mean (*n* = 5). * *p* < 0.05.

We then investigated the impact of gemigliptin on the activity of DPP-4 in both the salivary gland and serum, as well on levels of GLP-1. The results showed a significant reduction in both salivary gland and serum DPP-4 activity in the gemigliptin-treated group relative to the D-gal group. (Figure 2A,B). Likewise, there was a significant decrease in both salivary gland and serum DPP-4 levels in the gemigliptin group relative to the D-gal group (Figure 2C,D). GLP-1 levels in the salivary glands and serum were significantly higher in the gemigliptin-treated group than in the D-gal group, suggesting potential augmentation of the GLP-1-mediated effects (Figure 2E,F). These findings suggest that gemigliptin effectively reduces increased DPP-4 activity in both the salivary glands and serum of aging rats, resulting in an elevation of GLP-1 levels.

### 3.3. Gemigliptin Inhibits Apoptosis in the Salivary Glands of D-Gal-Injected Rats

**Figure 3:**
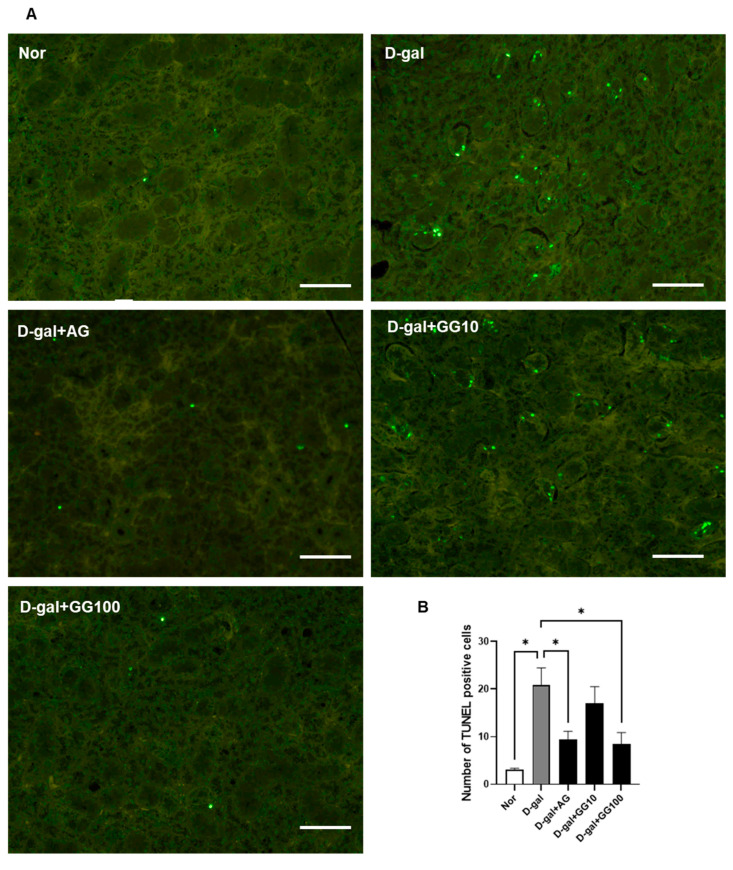

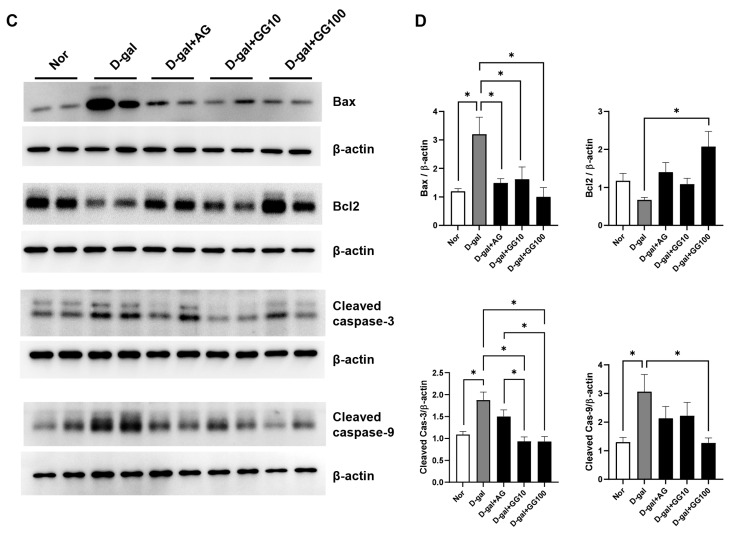
TUNEL assay and western blot of apoptosis-related proteins in salivary gland tissue. (**A**) TUNEL staining of apoptotic cells in the salivary gland sections (scale bar = 50 μm). (**B**) Quantitative analysis of TUNEL-positive cells in the salivary glands. (**C**) Effect of GG on Bax, Bcl2, caspase-3 and caspase-9 protein expression. (**D**) Optical density values of the protein bands from the western blot statistically quantified. β-actin was used as an internal control. All data are presented as the mean ± standard error of the mean (*n* = 5). * *p* < 0.05.

A TUNEL assay and western blot analyses were conducted to confirm the potential of gemigliptin to alleviate apoptosis in the salivary glands of rats treated with D-gal. The TUNEL assay revealed a significant increase in TUNEL-positive apoptotic cells in the D-gal group, while in the gemigliptin-treated group there was a concentration-dependent decrease in these cells. Additionally, the AG 100 mg/kg group also showed a significant decrease in TUNEL-positive cells (Figure 3A,B). Western blot data reflected altered expression profiles of apoptosis-associated proteins in gemigliptin-treated samples relative to the D-gal-exposed glands. In the D-gal group, we noted an increase in the expression of pro-apoptotic proteins Bax, cleaved caspase-3, and cleaved caspase-9, accompanied by a downregulation in the expression of the anti-apoptotic protein Bcl-2. In the D-gal group, the levels of pro-apoptotic proteins, such as Bax, cleaved caspase-3, and cleaved caspase-9, increased. Additionally, there was a decrease in the expression of the anti-apoptotic protein Bcl-2. Conversely, after administering gemigliptin, expression of the anti-apoptotic protein Bcl-2 increased in a concentration-dependent manner. Simultaneously, the expression of the apoptosis-promoting proteins Bax, cleaved caspase-3, and cleaved caspase-9 showed a concentration-dependent decrease (Figure 3C,D). As a result, it was confirmed that gemigliptin inhibits D-gal-induced salivary gland apoptosis.

### 3.4. Gemigliptin Inhibits the Expression of Oxidative Stress Markers in the Salivary Glands of D-Gal-Injected Rats

**Figure 4:**
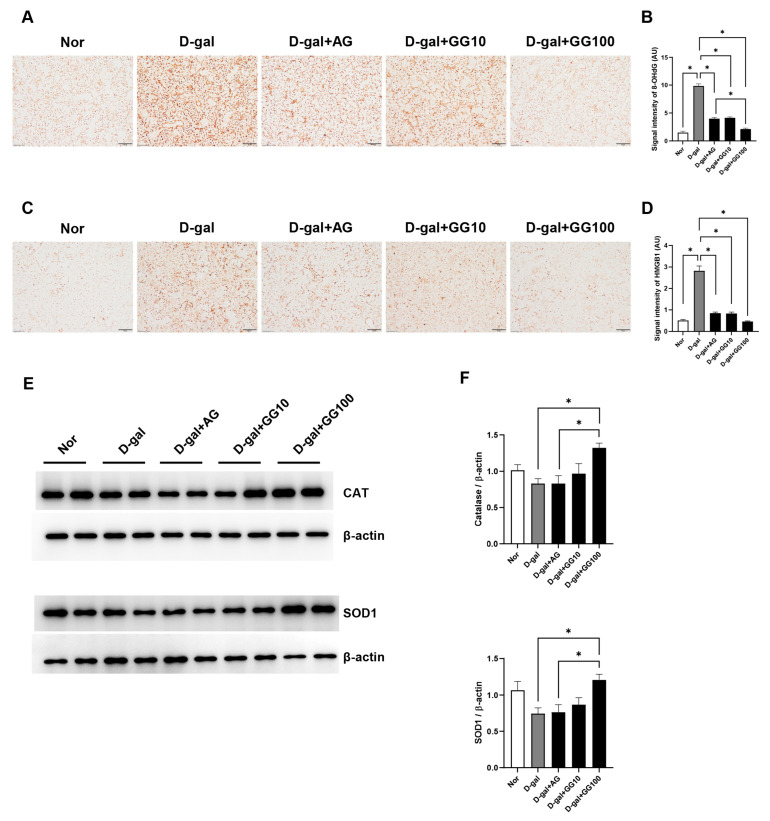
Effect of GG on ROS production in the salivary gland of D-gal-treated rats. (**A**) Immunohistochemical analysis of 8-OHdG expression performed in the salivary glands (scale bar = 50 μm). (**B**) Quantification of the 8-OHdG signal intensity. (**C**) Immunohistochemical analysis of HMGB1 expression performed in the salivary glands (scale bar = 50 μm). (**D**) Quantification of the HMGB1 signal intensity. (**E**) The effect of GG on the expression of Catalase (CAT) and Superoxide Dismutase 1 (SOD1) proteins measured using western blotting. (**F**) The optical density values of the protein bands from the western blot statistically quantified. β-actin was used as an internal control. All data are presented as the mean ± standard error of the mean (*n* = 5). * *p* < 0.05.

Protein levels were observed using western blot and immunohistochemistry (IHC) to confirm the antioxidant effects of gemigliptin in the salivary glands, staining. IHC was conducted to assess the extent to which 8-OHdG and HMGB1 had accumulated as markers of cellular damage induced by oxidative stress. The presence of 8-OHdG was confirmed in the salivary glands. Accumulation of 8-OHdG had increased in the D-gal group, but was reduced after AG and gemigliptin treatment. Furthermore, the reduction in 8-OHdG accumulation was greater in the gemigliptin 100 mg/kg group relative to both the AG and D-gal groups (Figure 4A,B). Similarly, the increased accumulation of HMGB1 in the D-gal group decreased with AG and gemigliptin treatment. A more pronounced reduction was observed in the gemigliptin 100 mg/kg group than the AG group (Figure 4C,D). Additionally, western blot analysis was performed to analyze markers of oxidative stress. The expression of antioxidant markers, CAT and SOD1, which had decreased in the salivary glands of D-gal-treated rats, increased with both AG and gemigliptin treatment. Notably, the group treated with gemigliptin at a dosage of 100 mg/kg exhibited a more significant increase in these markers than the group treated with AG (Figure 4E,F). These results suggest that gemigliptin can effectively suppress oxidative stress induced in the salivary glands of aging rats treated with D-gal.

### 3.5. Gemigliptin Reduces AGE Accumulation and RAGE Expression in the Salivary Glands of D-Gal-Injected Rats

**Figure 5:**
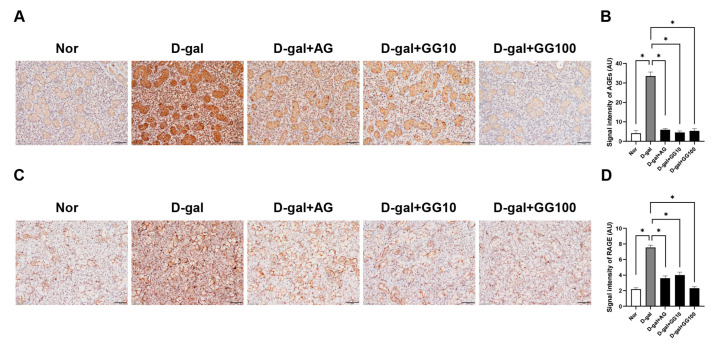

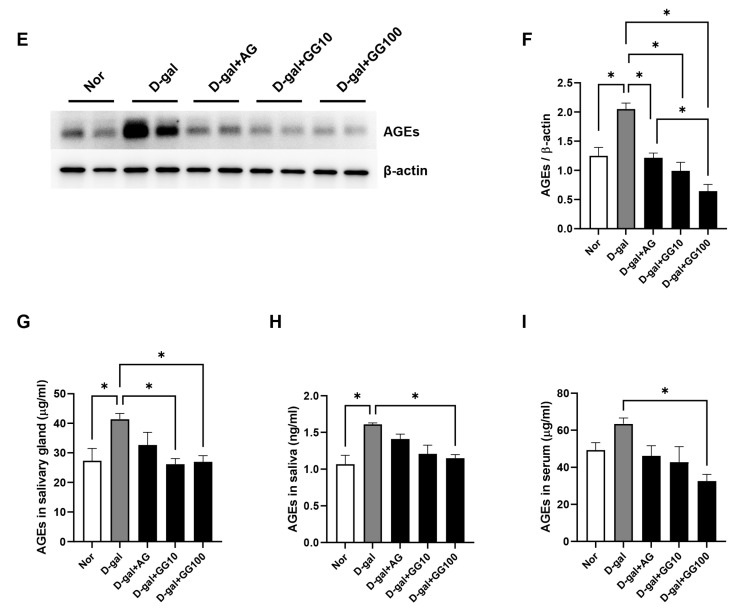
Effect of GG on the accumulation of advanced glycation ends (AGEs) in the salivary gland of D-gal-treated rats. (**A**) Immunohistochemical analysis of AGE expression performed in the salivary glands (scale bar = 50 μm). (**B**) Quantification of AGEs signal intensity. (**C**) Immunohistochemical analysis of RAGE expression performed in the salivary glands (scale bar = 50 μm). (**D**) Quantification of the RAGE signal intensity. (**E**) The effect of GG on the expression of AGEs measured using western blotting. (**F**) The optical density values of the protein bands from the western blot statistically quantified. β-actin was used as an internal control. The levels of AGEs were measured using an ELISA kit. (**G**) Measurement of serum AGE concentration in the salivary gland. (**H**) Measurement of serum AGE concentration in saliva. (**I**) Measurement of serum AGE concentration in serum. All data are presented as the mean ± standard error of the mean (*n* = 5). * *p* < 0.05.

IHC and western blot were performed to assess the accumulation of AGEs and the receptor for advanced glycation end products (RAGE) in the salivary glands, and in this manner investigate the impact of gemigliptin on the accumulation of AGEs induced by D-gal treatment. It was observed that the increased accumulation of AGEs in the D-gal group was reduced with both AG and gemigliptin treatments (Figure 5A,B). Similarly, IHC results for RAGE showed that the increased expression of RAGE in the D-gal group decreased after gemigliptin treatment (Figure 5C,D). Furthermore, western blot experiments were conducted to analyze the expression of AGEs, and the results were consistent with those obtained from IHC analysis; AGEs increased in the D-gal group but decreased with AG and gemigliptin treatment, with the most significant reduction observed in the gemigliptin 100 mg/kg group (Figure 5E,F). AGE levels were also measured in the salivary glands, serum, and saliva. AGE levels in the salivary glands increased in the D-gal group and decreased with both AG and gemigliptin treatment. Analysis of AGE levels in saliva also showed a decrease with AG and gemigliptin treatment, while increased levels were observed in the D-gal group. Similarly, in the serum, AGE levels increased in the D-gal group but decreased in a concentration-dependent manner with AG and gemigliptin treatment. These results suggest that gemigliptin effectively reduced the accumulation of AGEs in the salivary glands, saliva, and serum of rats treated with D-gal, and decreased the expression of RAGE in the salivary glands.

### 3.6. Gemigliptin Increases AQP5 Expression and Mucin Secretion in the Salivary Glands of Rats Injected with D-Galactose

**Figure 6:**
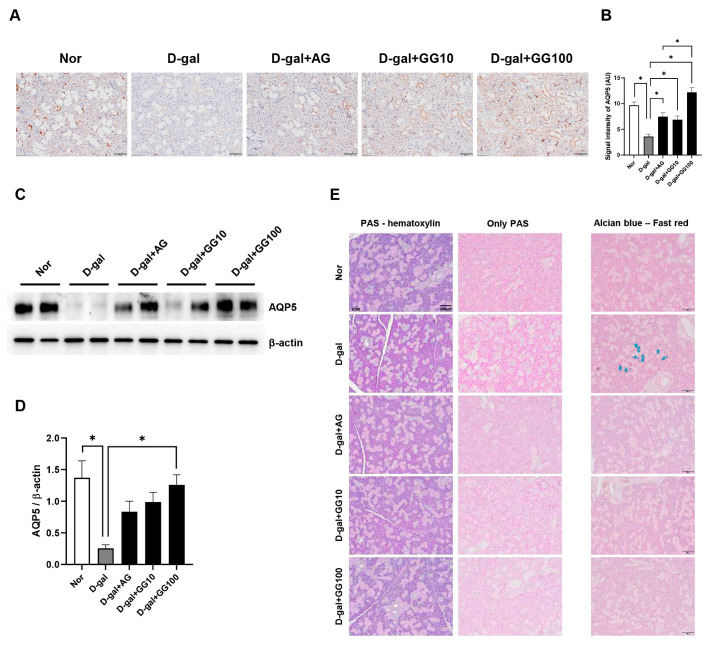
Effects of GG on mucin accumulation and AQP5 expression in the salivary gland of D-gal-injected rats. (**A**) Immunohistochemical analysis of AQP5 expression performed in the salivary glands (scale bar = 50 μm). (**B**) Quantification of AQP5 signal intensity. (**C**) Effect of GG on the expression of AQP5 as measured with western blotting. (**D**) The optical density values of the protein bands from the western blot were statistically quantified. β-actin was used as an internal control. (**E**) Periodic acid-Schiff (PAS) staining of salivary glands and Alcian blue (AB) staining of salivary glands (scale bar = 100 μm). All data are presented as the mean ± standard error of the mean (*n* = 5). * *p* < 0.05.

IHC was performed to confirm the aquaporin (AQP5) expression in the salivary glands. AQP5 expression was measured in both the salivary glands. The decreased expression of AQP5 in salivary glands of the D-gal group increased in a concentration-dependent manner when treated with gemigliptin (Figure 6A,B). Western blot analysis confirmed the protein expression of AQP5 in salivary gland lysates. The results were consistent with the AQP5 IHC findings, as the expression of AQP5 significantly decreased in the D-gal group and showed a concentration-dependent enhancement upon treatment with gemigliptin (Figure 6C,D).

PAS and AB staining were performed to confirm changes in the accumulation of neutral and acidic mucus in salivary gland acinar cells. In the normal group, the acini of salivary glands showed moderate staining when treated with AB and PAS reagents; however, in the D-gal group relatively intense staining was evident, suggesting an increased accumulation of both acidic and neutral mucus within the acini (Figure 6C,D). PAS staining intensity was relatively strong in the acini of salivary glands in the D-gal group, but weakened in the gemigliptin-treated group (Figure 6E). This suggests a more significant neutral mucin blockade in the D-gal group than in the gemigliptin-treated group. Relative to the normal group, AB staining intensity was relatively strong in the D-gal group but weak in the gemigliptin 100 mg/kg group. Interestingly, in the D-gal group, some acini in the salivary glands exhibited strong positive AB staining (Figure 6E). These results showed that the treatment with gemigliptin in D-gal-treated rats resulted in an increase in AQP5 expression in the salivary glands and a decrease in the accumulation of neutral and acidic mucins in the acini.

## 4. Discussion

Dry mouth is now among the most common issues faced by older adults. Saliva plays a crucial role in maintaining oral health and overall quality of life [1]. Reduction in saliva flow and changes in saliva composition can lead to tissue damage in the oral cavity, rendering it susceptible to oral-related diseases [24]. Similar to natural aging models, the D-gal-induced aging model exhibits neurological damage, decreased activity of antioxidant enzymes, and impaired immune responses [25]. Accumulation of AGEs in the salivary glands of D-gal-induced aging rats results in the generation of additional ROS, loss of acinar cells, and an increase in cell apoptosis [9]. In this study, we investigated the effects of gemigliptin, a DPP-4 inhibitor, induced by D-gal on salivary gland function in aging rats.

In the present study, we observed an increase in AGE accumulation and a decrease in saliva secretion in the salivary glands of aging rats. Various previous studies have reported that oxidative stress and AGEs play significant roles in the aging process. The increased levels of AGEs induced by D-gal can interact with RAGE, leading to aging, mitochondrial dysfunction, oxidative stress, cellular damage, and inflammation [26]. When excessive D-gal accumulates in the body, AGEs form by binding to proteins or peptides [27]. Numerous studies have shown that the D-gal-induced aging model replicates natural aging and is closely related to real-world aging-related processes [28,29]. In our experiments, we confirmed an increase in AGEs accumulation and ROS induced by D-gal. Previous research has provided evidence that the inhibition of AGE formation and oxidative stress in D-gal-induced aging disease models alleviates the condition [30,31]. We further reported an improvement in salivary gland function through physical exercise in the D-gal-induced aging rat model. This study highlighted how the inhibition of AGE formation can induce the enhancement of salivary gland function [9]. Our results also showed that the accumulation of AGEs and ROS in aging rats is reduced by the administration of gemigliptin, a potent anti-glycation agent. Furthermore, AG, a well-known inhibitor of AGEs, has been shown to reduce the accumulation of AGEs in salivary glands. These findings underscore the significant role of glycation as a major contributor to D-gal-induced aging [32]. Therefore, our study suggests that gemigliptin contributes to the improvement of salivary gland function in aging rats through the suppression of AGE accumulation and ROS generation.

Our results showed an increase in DPP-4 activity and a decrease in GLP-1 levels in the salivary glands and serum of aging rats. However, when gemigliptin was administered, DPP-4 activity was reduced and GLP-1 levels rose. In animal models of diabetes and hyperglycemia, GLP-1 exhibits antioxidant effects on endothelial cells [33]. Pharmacologically, DPP-4 inhibition is associated with a reduction in oxidative stress [34]. DPP-4 inhibitors have been shown to have a protective effect in conditions characterized by impaired insulin signaling and oxidative stress induced by D-gal in aging rats [16]. Additionally, DPP-4 deficiency has been reported to lead to lower levels of AGEs and improved age-related complications [35]. Increased GLP-1 in DPP-4 deficient mice has been shown to regulate AGE formation and improve diabetic nephropathy [36]. There are also reports of improved GLP-1 levels in D-gal-induced aging mice through antioxidant actions [37]. We observed an increase in DPP-4 activity, a decrease in GLP-1 levels, and elevated AGE accumulation in aging rats. These factors subsequently induced ROS and, ultimately, apoptosis in salivary gland cells.

According to previous research, the increased ROS induced by D-gal leads to dysfunction in the salivary glands. This dysfunction can be improved by inhibiting ROS [9,38]. There are also reports of gemigliptin improving salivary gland dysfunction in diabetic rats [23]. As a result, D-gal induces ROS-induced apoptosis in salivary glands, while gemigliptin effectively suppresses ROS through DPP-4 inhibition, increased GLP-1 levels, and reduced AGE accumulation, in due course exerting a protective effect on salivary gland cells.

We observed morphological differences in the ratio of acinar cells to ductal cells in the salivary glands. Relative to the control group, aging rats showed a decrease in the proportion of acinar cells and an increase in ductal cells in their salivary glands. Similar results have been observed in diverse salivary gland dysfunction models. The age-related reduction in secretory cells in salivary glands is thought to be the result of decreased cell proliferation or increased cell death [39]. Consistent with our results, age-related reduction in acinar cell area has also been observed in human salivary glands [40]. Salivary gland dysfunction-induced mice showed a decrease in acinar area and an increase in duct area in both male and female mice [41,42]. Likewise, our results showed that the ratio of acinar cells increased following the improvement of salivary gland dysfunction by gemigliptin. We also measured salivary flow after administering pilocarpine, a parasympathetic agonist on muscarinic acetylcholine receptors in salivary glands [43]. The decrease of salivary secretion induced by D-gal increased with the administration of gemigliptin.

The increase in ROS-induced apoptosis caused by D-gal leads to salivary gland dysfunction. Decreased saliva flow is associated with salivary dysfunction, and α-amylase in saliva is the most abundant protein responsible for starch hydrolysis, serving as an indicator of salivary gland function evaluation [44]. Improvements in salivary gland function were observed in aged rats and were associated with an increase in amylase levels [45]. Our results showed a decrease in saliva flow and amylase levels in the saliva of aging rats. In contrast, amylase levels decreased in the salivary glands of aging rats. Oxidative stress, caused by the accumulation of ROS, altered secretion functions, such as saliva secretion, salivary amylase, and calcium levels, in the salivary glands of rodents [46]. Consequently, the administration of gemigliptin in aging rats resulted in an increase in saliva flow and amylase levels in saliva, while levels of amylase in the salivary glands decreased. These results suggest potential issues in the secretion process of amylase from the salivary glands.

We also examined the accumulation of acidic and neutral mucins in the salivary glands of rats. One of the primary functions of saliva, which consists of various compounds, is to provide mucin-based lubrication to mucosal surfaces [47]. While no changes were observed in the size of the acini, our results confirmed an increased accumulation of mucins in the salivary glands of aging rats, and a reduction in the amount accumulated with gemigliptin treatment. Therefore, our findings suggest that gemigliptin reduces the accumulation of mucins in acini, in this manner contributing to the improvement of salivary gland dysfunction. In diabetic animal models, stronger AB and PAS staining densities in acini indicate a higher level of mucin accumulation in diabetic salivary glands acini [11,48]. Saliva formation consists of two stages. In the primary secretion, progenitor cells produce and secrete the majority of the primary fluid in saliva [49]. In our experiment, we observed the increased accumulation of mucins in the salivary glands and a reduced concentration of α-amylase in saliva in the D-gal group. These results suggest that D-gal-induced salivary gland dysfunction may have more to do with secretory disorders rather than disorders affecting saliva production. The submandibular gland of rats is a mixed seromucous gland. In this study, both AB and PAS staining showed that AB- and PAS-positive mucin was highly accumulated in salivary glands of db/db mice. Huang et al. also showed that acini of submandibular glands were moderately stained by AB and PAS reagents in normal db/m mice but strong staining was observed in db/db mice with hyperglycemia. Stronger AB and PAS staining densities of acini in db/db mice demonstrated that more mucins accumulated in the acini of salivary glands. Therefore, they can reflect changes in salivary glands functions. Accumulation of mucins in salivary glands demonstrated that secretory functions of salivary glands were impaired [48]. Additional experiments would be needed to provide further evidence that confirms this hypothesis.

Taking into consideration the significant role of AQP5 in salivary secretion, we then examined the expression level of AQP5 in the salivary glands. AQP5 is a channel protein that regulates the movement of water through the apical membrane of the secretory and absorptive cells in response to osmotic gradients. It plays an essential functional role in salivary secretion in the salivary glands of mice [50]. Mice lacking AQP5 exhibit a 60% reduction in pilocarpine-induced salivary secretion. Moreover, AQP5-null mice show a significant reduction (greater than 60%) in salivary gland water permeability, indicating the crucial role of AQP5 in water permeability and salivary secretion in acinar cells [51]. We observed a decrease in AQP5 expression in the salivary glands of aging rats relative to the normal group. Upregulating AQP5 can improve secretion dysfunction [10,52], and the administration of gemigliptin increased the expression of AQP5 in the salivary glands of aging rats in a concentration-dependent manner.

**Figure 7:**
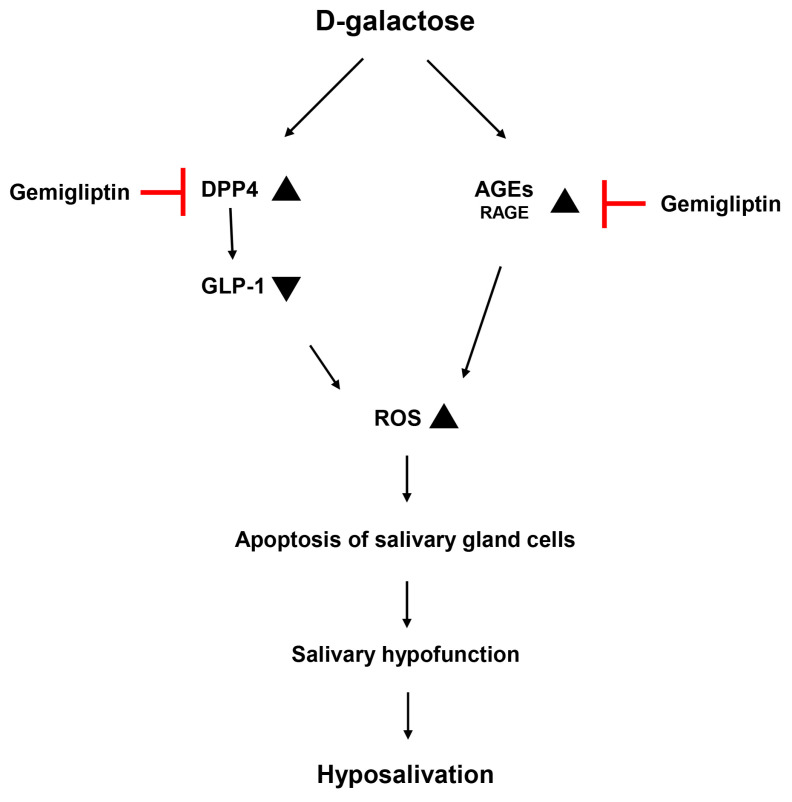
Possible mechanisms of action of GG on salivary glands with D-gal-induced hypofunction.

In conclusion, gemigliptin administered to aging rats alleviated ROS-induced apoptosis by inhibiting DPP-4 activity and suppressing AGE accumulation. These cytoprotective effects improved salivary gland function by increasing salivary flow rate and amylase levels in saliva, reducing mucin accumulation in the salivary glands, and upregulating AQP5 (Figure 7). Gemigliptin may be a potent agent for patients with diabetes-related salivary gland dysfunction.

## Figures and Tables

**Table 1:** Chemical structures and pharmacological characteristics of DDP-4 inhibitors.

	Gemigliptin	Saxagliptin	Linagliptin	Vildagliptin	Alogliptin
Formula	C_18_H_19_F_8_N_5_O_2_	C_18_H_25_N_3_O_2_	C_25_H_27_N_8_O_2_	C_17_H_25_N_3_O_2_	C_18_H_21_N_5_O_2_
Chemical structure	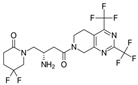	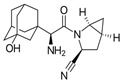	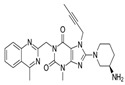	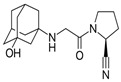	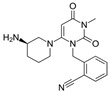
Therapeutic dose	50 mg/day	5 mg/day	5 mg/day	100 mg/day	25 mg/kg
DPP-4 inhibition IC_50_	6.3 nmol/L	50 nmol/L	1 nmol/L	62 nmol/L	24 nmol/L

## Data Availability

The data presented in this study are available on request from the corresponding author.
